# Immunobiological Characteristics of the Attenuated African Swine Fever Virus Strain Katanga-350

**DOI:** 10.3390/v14081630

**Published:** 2022-07-26

**Authors:** Alexey D. Sereda, Mikhail E. Vlasov, Galina S. Koltsova, Sergey Y. Morgunov, Dmitry A. Kudrjashov, Irina P. Sindryakova, Olga L. Kolbasova, Valentina M. Lyska, Andrei Y. Koltsov, Sergey P. Zhivoderov, Elena Y. Pivova, Vladimir M. Baluishev, Andrey E. Gogin, Denis V. Kolbasov

**Affiliations:** Federal Research Center for Virology and Microbiology (FRCVIM), Petushki District, Vladimir Region, 601125 Volginsky, Russia; vlasovmikhail1993@yandex.ru (M.E.V.); lila5757@yandex.ru (G.S.K.); dohliy55555@mail.ru (S.Y.M.); info@ficvim.ru (D.A.K.); sindryakova.irina@yandex.ru (I.P.S.); olgakolbasova@gmail.com (O.L.K.); vliska@yandex.ru (V.M.L.); kolcov.andrew@gmail.com (A.Y.K.); zhivoderov-serg@mail.ru (S.P.Z.); lenamail09@inbox.ru (E.Y.P.); balyshevvm@rambler.ru (V.M.B.); kolbasovdenis@gmail.com (D.V.K.)

**Keywords:** African swine fever virus, seroimmunotype, genotype, attenuated strains, immunobiological characteristics, protective immunity

## Abstract

The African swine fever virus (ASFV) is the cause of a recent pandemic that is threatening the global pig industry. The virus infects domestic and wild pigs and manifests with a variety of clinical symptoms, depending on the strain. No commercial vaccine is currently available to protect animals from this virus, but some attenuated and recombinant live vaccine candidates might be effective against the disease. This article describes the immunobiological characteristics of one such candidate—the laboratory-attenuated ASFV strain, Katanga-350—which belongs to genotype I. In this study, we assessed clinical signs and post-mortem changes, the levels of viremia and the presence of viral DNA caused by injection of ASF virus strains Katanga-350, Lisbon-57, and Stavropol 08/01. Intramuscular injection of this strain protected 80% of pigs from a virulent strain of the same genotype and seroimmunotype (Lisbon-57). At least 50% of the surviving pigs received protection from subsequent intramuscular infection with a heterologous (genotype II, seroimmunotype VIII) virulent strain (Stavropol 08/01). Virus-specific antibodies were detectable in serum and saliva samples between 8–78 days after the first inoculation of the Katanga-350 strain (the observational period). The results suggested that this strain could serve as a basis for the development of a recombinant vaccine against ASF viruses belonging to seroimmunotype I.

## 1. Introduction

African swine fever (ASF) is a contagious, hemorrhagic, notifiable disease of swine. Due to the unavailability of an ASF vaccine, it causes serious economic losses worldwide [1].

ASF is caused by a large, enveloped, intricately structured, double-stranded DNA virus that belongs to the genus Asfivirus of the family Asfarviridae [2].

African swine fever was first reported in East Africa in 1921 [3]. Subsequently, ASF cases have been reported in most Sub-Saharan countries [4,5]. In the late 1950s, ASF was reported in Europe for the first time. The virus was introduced to Portugal from Angola [6]. From the 1960s to the 1980s, the ASFV emerged in Western Europe several times (Spain, the Netherlands, France, Italy, etc.), in the Soviet Union (the Odessa region) and even in a few countries in South America (the Caribbean and Brazil). During this period, some attempts were made to broadly develop and even to implement an attenuated vaccine. But this led to a wider spread of the virus. Nonetheless, by the mid-1990s, ASF had been eradicated in Europe and America, excluding Sardinia, Italy [7,8,9]. In 2007, the disease was unexpectedly reported in the Republic of Georgia, and then it spread widely in Eastern Europe and some countries of Western Europe [10,11,12,13]. In 2018, ASF was introduced to China for the first time. Since then, it has spread to other parts of Asia, including Vietnam, Cambodia, North Korea, Laos, Indonesia, Myanmar, the Philippines and South Korea [14,15,16,17,18].

Despite all efforts made since the 1950s to contain ASFV, there is currently no available effective and safe vaccine against it. Disease outbreaks in domestic pigs are currently controlled by complex and strict quarantine measures that include total depopulation and animal movement bans. Prospects for the development of a means of specific ASF prevention are associated with live attenuated or recombinant vaccines [19]. Attenuated or low pathogenic ASFV strains could elicit protective immune responses to homologous or even heterologous virulent strains in swine [20,21], providing sterile immunity in some cases [22]. However, a significant proportion of the vaccinated pigs developed unacceptable post-vaccination clinical symptoms such as pneumonia, movement disorders (dystaxia), skin ulcers, abortion, and mortality [23,24].

A series of studies on the selection of attenuated strains for the development of live vaccines against ASF were conducted at the Federal Research Center for Virology and Microbiology (FRCVM), Russia from the 1980s to the 2000s. According to the adopted concept, live vaccines were developed for temporal protection of pigs at large ASF-affected pig farms meant for subsequent planned slaughtering and processing for cooked meat products. For these live vaccines, attenuated candidate strains must have low or moderate reactogenicity, be unreversible, cause level- and time-limited viremia, not cause autoimmune disorders, and not be transmittable from vaccinated to naïve pigs kept together. As a result of these requirements, only several candidate strains for vaccines against ASFV seroimmunotypes I–V were obtained [25]. For a long time, there was a problem with obtaining an attenuated strain for protection against ASFV seroimmunotype I isolates. Eventually, the attenuated strain, LK-111, was obtained by selective passaging of the ASFV reference virulent strain Lisbon-57 (seroimmunotype I) in primary pig bone marrow cells (PBMC) and peripheral blood lymphocyte culture systems (PBLS). Because of its low protective ability, prolonged viremia and autoimmune complications in inoculated pigs, this strain was not recommended as a vaccine candidate [26]. Attenuated variants of Diamant, Kimakia, Madeira-65 and Katanga-105 isolates had similar disadvantages [25]. Later, the attenuated hemadsorbing strain, Katanga-350, was obtained by passaging of the virulent ASFV strain, Katanga (seroimmunotype I), in a PBMC cell culture. According to the preliminary results, this strain met the requirements for vaccines mentioned above and was considered as a main candidate for the production of protective preparations against ASF viruses belonging to seroimmunotype I [27].

The aim of this study was to provide insight into the immunobiological characteristics of the ASFV strain, Katanga-350, attenuated under laboratory conditions and to assess the possibility of using it as a vaccine candidate.

## 2. Materials and Methods

### 2.1. Viruses and Cells

The following ASFV strains were received from the collection of microorganisms of the Federal Research Center for Virology and Microbiology (FRCVIM, Volginsky, Russia): Lisbon-57, Congo-49, Mozambique-78, France-32, TSP-80, TS-7, Uganda, Stavropol 08/01, Davis, Katanga, Katanga-350 [25].

The infectious activities of these strains were determined by titration in PBLS (four wells for each tenfold dilution) [28]. The results were examined by the presence of hemadsorption phenomenon after 5–7 days. The virus titers were calculated according to the method described by B.A. Kerber in I.P. Ashmarin’s modification and expressed in 50% hemadsorbing units per mL (HAU_50_/mL) [29].

### 2.2. Animal Experiments and Ethics Statement

Two- to three-month-old female and male (20–30 kg) pigs of the Large White pig breed purchased from the Experimental Animal Preparation Sector of the FRCVM were used in the experiment. They were housed in a BSL3-Ag laboratory of the FRCVIM. There was a 6-day acclimation period before commencement of the study. During this period, pigs were trained to chew on the ropes used to collect oral fluid samples.

The pigs were kept and euthanized in accordance with the AVMA Guidelines for the care and use of laboratory animals [30], and all efforts were made to minimize suffering.

Nine pigs were randomly divided into three groups housed in three separate premises. There were five pigs in Group 1. Groups 2 and 3 (control) consisted of two pigs each kept individually. Pigs in Group 1 (## 1–5) were inoculated intramuscularly with the attenuated ASFV strain, Katanga-350, at a dose of 10^6.0^ HAU_50_ twice (on day 0 and day 14). Groups 1 (pigs ## 1–5) and 2 (pigs # 6 and # 7) were challenged intramuscularly with the virulent ASFV strain, Lisbon-57, at a dose of 10^3.0^ HAU_50_ on the 28^th^ day. Groups 1 (pigs ## 2–4) and 3 (pigs # 8 and # 9) were challenged intramuscularly with the virulent ASFV strain, Stavropol 08/01, at a dose of 10^3.0^ HAU_50_ on the 42^nd^ day.

Blood specimens from the anterior vena cava were sampled at different days post inoculation (dpi) and post challenging (dpc) with the strains Lisbon-57 and Stavropol 08/01 (dpc-L, dpc-St, respectively) and collected in test tubes (10 mL each) with coagulant to receive the serum and with anticoagulant (lithium heparin) to determine the viremia levels [31].

### 2.3. Preparation of Peripheral Blood Leukocytes

Heparinized blood samples collected from the anterior vena cava were layered on a Ficoll–Hypaque gradient (density 1077 g/cm^3^, GE Healthcare, USA) and centrifuged (22 °C, 400 g, 30 min). Cells from the interphase were collected and washed out with Hanks balanced saline solution three times by centrifugation (4 °C, 400 g, 5 min). As a growth medium (pH 7.60–7.65), we used 0.1% lactalbumin hydrolysate in Earle’s saline solution with 10% donor pig blood serum. PBLS primary cell culture was distributed in 48-well plastic micropanels (Nunc, Denmark) with a working volume of 1.0 mL. Wells were filled with cell suspension to achieve a concentration of 3.0–3.5 million cells/mL. Micropanels were incubated in a CO_2_ incubator and under the following conditions: CO_2_ concentration 5%, relative humidity 90%, temperature (37.0 ± 0.5) °C.

### 2.4. Assessment of Hemadsorbtion Inhibition (HAdI)

The following strains of ASFV were used in the experiment: Lisbon-57, Congo-49, Mozambique-78, France-32, TSP-80, TS-7, Uganda, Stavropol 08/01, Davis, Katanga and Katanga-350. All viral strains were refreshed (1–2 passages) in primary PBLS cell cultures before the experiment. The average infectious activity of the viruses was 10^6.5^–10^7.5^ HAU_50_/mL.

The following sera were used in the experiment HAdI: serotype-specific (I-IX serotypes) pig sera with an activity of 1:80–1:160 HAdI and normal swine serum heat-treated for 30 min at 56 °C. After 2 days, detached and excess erythrocytes were removed from the microplates. For this purpose, the microplates were shaken for 5–15 s on a shaker device (6–8 vibrations/s) and then the suspension was removed. Then, 0.9 mL of the growth medium was added to each well, followed by 0.05 mL of the virus with an infectious titer of 10^4.0^ HAU_50_/mL mixed with 0.05 mL of serotype-specific or normal serum. The microplates were placed in a CO_2_ incubator.

To perform HAdI, the following controls were used: cell cultures with normal pig serum to assess the quality of cell culture; serotype I-IX-specific sera to control for the absence of nonspecific hemadsorption; and ASFV reference strains (I-IX seroimmunotypes) to control for the quality of hemadsorption. HAdI results were assessed after exposition for 48–72 h by well-explicit hemadsorption (at least 2–5 cells with specific hemadsorption in the field of vision of the microscope (400× magnitude objective)) in the control samples or by its absence in the controls with specific sera. Delayed hemadsorption of an isolate caused by the presence of one of the nine reference sera indicated that this specific isolate belonged to a certain serotype of the virus.

### 2.5. Sample Collection

To collect oral fluids, a cotton triple-stranded rope (TD PROMT LLC, Lipetsk, Russia) was suspended in a pen in front of each animal at shoulder height for 20–30 min for chewing. For this period of time the animals were kept individually. The pigs chewed on the ropes, and after that the oral fluids were collected individually by cutting off the wet end of the rope and putting it into a plastic bag (TD PROMT LLC, Lipetsk, Russia). Then the liquid was squeezed into a plastic vial (Fisher Scientific Company LLC, Pittsburgh, PA, USA) and centrifuged at 1200× *g* for 2 min. The supernatant was collected for testing.

Sera and whole blood samples were obtained by jugular venipuncture. To obtain whole blood, the blood samples were collected in EDTA tubes (TD VIK LLC, Lyubertsy, Russia), and to obtain blood serum, the whole blood was collected in serum tubes (TD VIK LLC, Lyubertsy, Russia), allowed to clot, and then centrifuged for 10 min at 2000× *g*.

Three aliquots of all the samples described above were prepared and stored in 5-mL or 2-mL cryovials at minus 40 °C until testing (Simport Scientific, Saint-Mathieu-de-Beloeil, QC, Canada) (Corning Inc, Corning, NY, USA). All equipment used for sampling was cleaned and disinfected between pigs and uses. All samples were frozen and thawed once prior to the test.

### 2.6. Polymerase Chain Reaction (PCR)

The viral DNA was extracted from all EDTA blood and saliva samples using the QIAmp_DNA Mini kit (QIAGEN, Hilden, Germany) according to the manufacturer’s instruction. Detection of ASFV genomic DNA was carried out, according to the protocol described by Fernandez-Pinero et al. (2013), on Bio-Rad CFX 96 Real-Time Detection Systems (Bio-Rad, Hercules, CA, USA) [32]. Samples with Ct (cycle threshold) < 45.0 were considered as positive, while samples with no Ct value were considered as negative.

### 2.7. DNA Sequencing and Analyses

DNAs from samples of infected cell culture were isolated using the DNeasy blood and tissue kit (Qiagen, Hilden, Germany) according to the manufacturer’s instructions. Analysis of viral genomic DNA was performed by PCR using primers and TaqMan probes for ASFV B646L gene detection, as presented by F.J. Haines (2013) [33]. For endogenous control of PCR, swine GAPDH gene detection was included in the analysis.

For genotyping of ASFV, the B646L gene encoding the p72 protein was used [34]. The EP153R and EP402R genes encoding the lectin C-type protein and CD2v protein respectively were used for serogroup clustering [35]. The PCR products were analyzed by electrophoresis in a 2% agarose gel in TAE buffer. The PCR products were purified using the Cleanup Standard kit (Evrogen, Moscow, Russia) and subjected to DNA sequencing. Sequencing was performed by the Sanger method on 3130xl Genetic Analyzer (Applied Biosystems, Waltham, MA, USA), and the obtained data were analyzed with the Sequence Analyzer software (Applied Biosystems, Waltham, MA, USA). The ClustalW algorithm was used for multiple sequence alignment [36]. Phylogenetic trees were constructed using the maximum likelihood method, in which phylogenetic distances were built according to Kimura’s two-parameter model (K2P) [37] using MEGA 7.0 software [38]. The nodes were determined via bootstrap analysis with 1000 replicates.

### 2.8. Detection of Anti-ASFV Antibodies

Serum samples were tested in duplicates using the INgezim PPA Compac solid-phase ELISA test kit (Ingenasa, Madrid, Spain) [39]. According to the kit instructions, the status of each tested serum was expressed using the coefficient of inhibition (x%). Oral fluid samples were tested using ID Screen^®^ African Swine Fever Oral Fluids Indirect expressed in OD_450_. [40].

### 2.9. Clinical Evaluation and Sampling

The severity of the disease was assessed by a quantitative clinical score (CS) obtained by adding the values for the following eight clinical signs recorded on a daily basis, as detailed by Gallardo et al. (2015, 2017): fever parameters, anorexia, recumbency, skin hemorrhage or cyanosis, joint swelling, respiratory distress, ocular discharge and digestive findings were assigned points on an ascending severity scale of 0–3. Pre-determined humane endpoints included a pig displaying severe signs of fever, anorexia, recumbence, respiratory distress and digestive signs for more than two consecutive days, or a total CS > 18 [41,42].

### 2.10. Statistical Analysis

Statistical analysis of the results was performed using multifactor analysis. Differences between counts were considered significant at *p* < 0.05.

## 3. Results

### 3.1. Nucleotide Sequencing and Phylogenetic Analysis of the Strain Katanga-350

**Figure 1:**
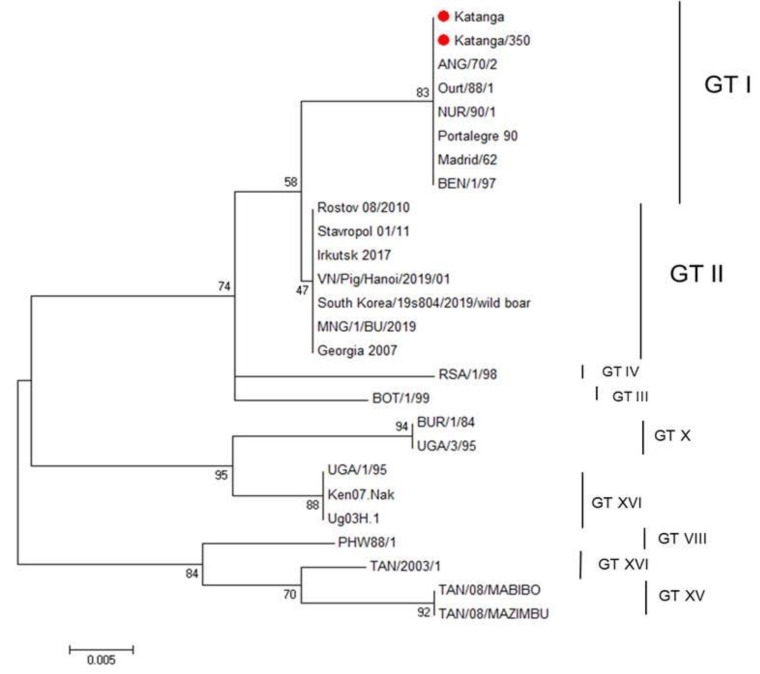
Maximum likelihood tree of ASFV B464L gene sequences (1000 bootstrapping replicates). Genotype status is indicated (GT#). The ASFV strains, Katanga and Katanga-350, are indicated with a red circle.

We used nucleotide sequencing and phylogenetic analysis to characterize the ASFV strain, Katanga-350. Initially, for genetic analysis of this ASFV strain, we amplified the B646L gene encoding the p72 protein. For comparative genomic analysis, reference genome sequences of different genotypes published in GenBank were added. The sequence of the B646L gene of the original virulent ASFV strain, Katanga (GenBank #KJ526355.1), had been determined earlier [35] and was also used for analysis. The ASFV strain, Katanga-350, was classified as belonging to genotype I, as was previously reported for the original virulent ASFV strain, Katanga (C-type lectin-like protein gene and CD2v gene—OP019316, p72 (B646L) gene—OP019315) (Figure 1).

**Figure 2:**
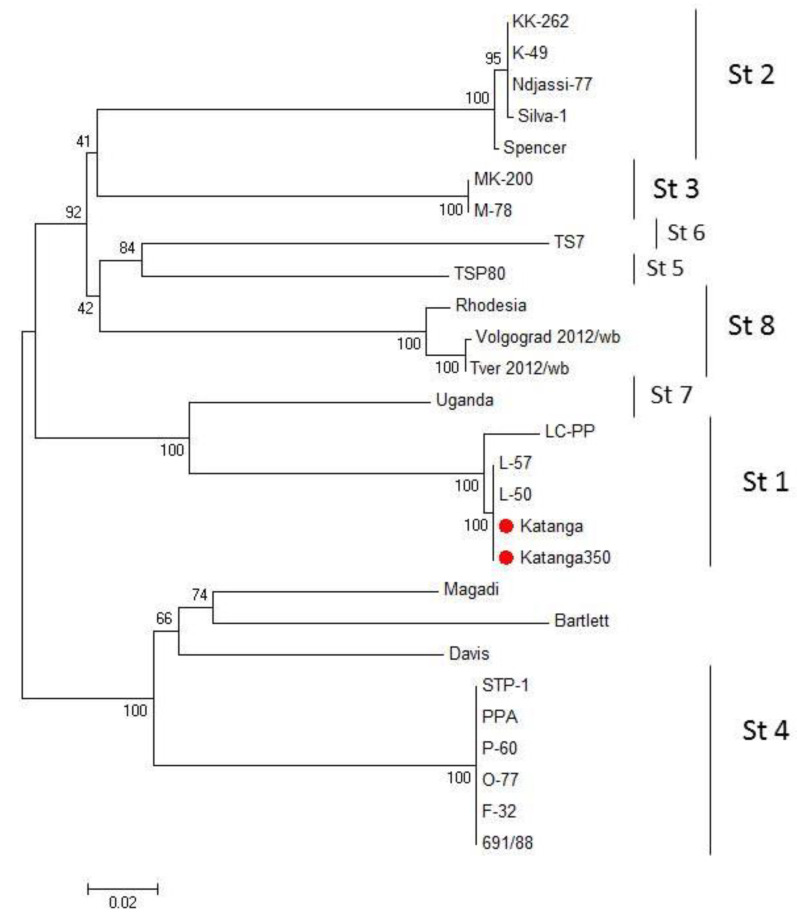
Maximum likelihood tree of ASFV CD2v and C-type lectin gene sequences (1000 bootstrapping replicates). Serotype status of typed viral taxa is indicated (St#). The ASFV strains, Katanga and Katanga-350, are indicated with a red circle.

We compared the EP153R and EP402R gene sequences of the ASFV for serotype classification [35]. A phylogenetic tree was constructed using the sequences of the EP153R and EP402R genes by the maximum likelihood method (Figure 2). Sequences from Katanga (GenBank #KM609340.1) and Katanga-350 were identical. Phylogenetic analysis based on these gene sequences revealed that the ASFV strain, Katanga-350, was grouped closely and reliably within the tree with the ASFV reference strain, Lisbon-57 (GenBank #KM609344.1), and other strains of serotype 1 (Figure 2). All sequences of the ASFV strain, Katanga-350, were deposited into GenBank (accession nos.).

### 3.2. Serotyping of ASFV Strains, Katanga, Katanga-350 and Stavropol 08/01

We considered it necessary to confirm the serotype specificity of the strains before the experiment. Virulent and attenuated ASFV strains taken after long-term storage were examined for their serotype characteristics. The results of HAdI are presented in Table 1.

The results confirm that the strains, Katanga and Katanga-350, belong to serotype I, while Stavropol 08/01 belongs to serotype VIII.

### 3.3. Protection of Pigs Inoculated with Katanga-350 Strain from Challenge with Virulent ASFV Isolates

Table 2 summarizes the results of the bioassay.

**Figure 3:**
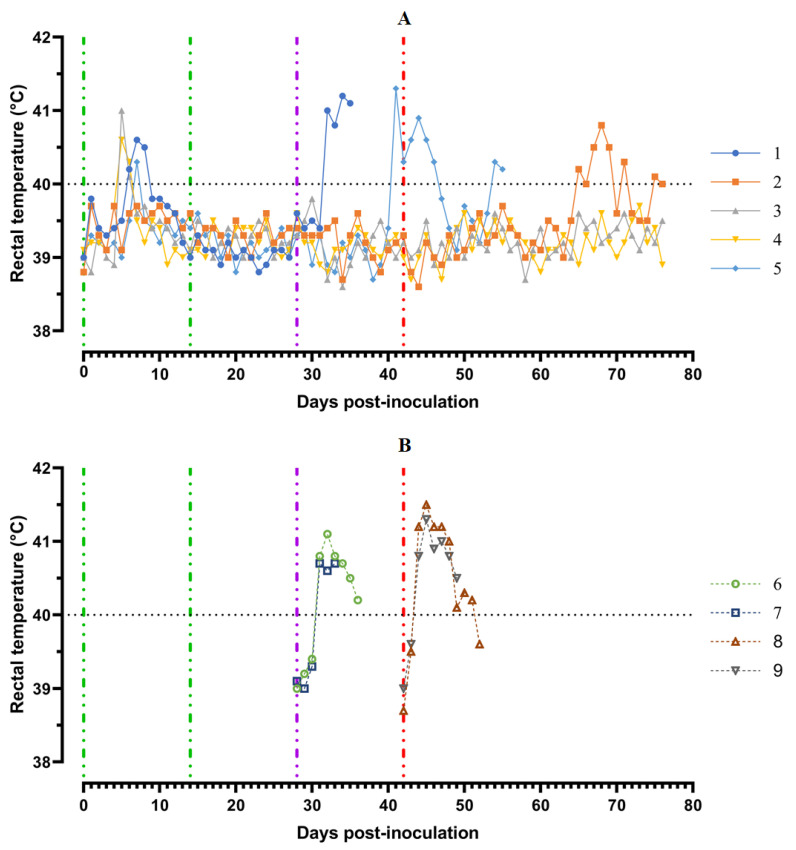
Kinetics of body temperature values in pigs: (**A**) group 1 (## 1–5) inoculated with 10^6.0^ HAU_50_ ASFV–Katanga-350 and challenged at a dose of 10^3.0^ HAU_50_ ASFV–Lisbon-57 and ASFV–Stavropol 08/01; (**B**) group 2 challenged with ASFV-Lisbon-57 (## 6, 7) and group 3 challenged with ASFV–Stavropol 08/01 (## 8, 9). Each curve represents an individual animal’s values. Vertical dashed lines: green—days of inoculation of ASFV-Katanga-350, purple—challenge by ASFV–Lisbon-57, red—infected by the ASFV–Stavropol 08/01 strain.

Pigs from Group 1 (## 1–5) were inoculated by the strain, Katanga-350, twice within an interval of 14 days. In the period from 5 to 8 dpi, the animals showed a slight manifestation of clinical signs of the disease along with hyperthermia (## 1, 3–5) (Figure 3).

**Figure 4:**
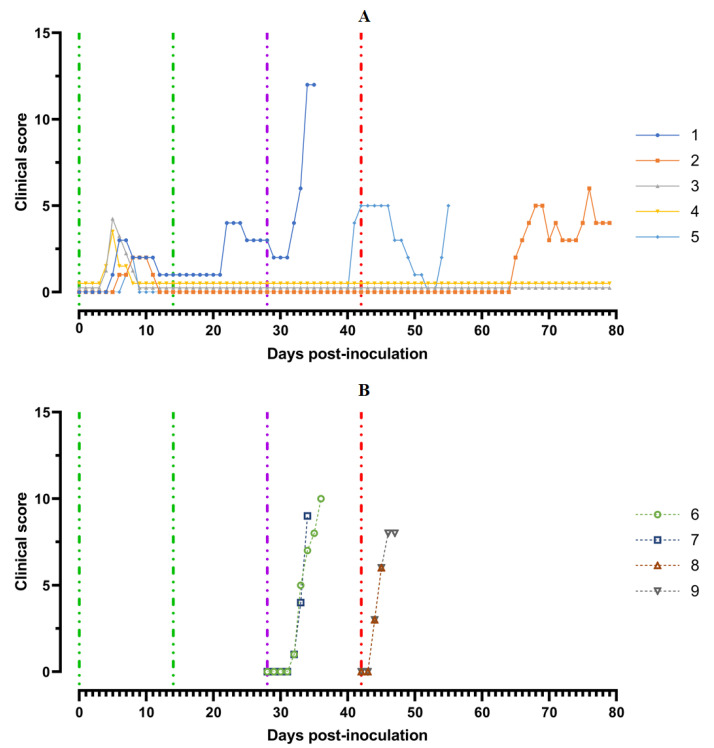
Clinical score of pigs: (**A**) group 1 (## 1–5) inoculated with 10^6.0^ HAU_50_ ASFV–Katanga-350 and challenged at a dose of 10^3.0^ HAU_50_ ASFV–Lisbon-57 and ASFV–Stavropol 08/01; (**B**) group 2 challenged with ASFV–Lisbon-57 (## 6, 7) and group 3 challenged with ASFV–Stavropol 08/01 (## 8, 9). Each curve represents an individual animal’s values. Vertical dashed lines: green—days of inoculation of ASFV–Katanga-350, purple—challenge by ASFV–Lisbon-57, red—infected by the ASFV–Stavropol 08/01 strain.

A body temperature higher than 40 °C was recorded in three pigs for 1–2 days, and in one animal (# 1) for 3 days. Along with hyperthermia, insignificant clinical signs were observed in the animals (Figure 4A).

**Figure 5:**
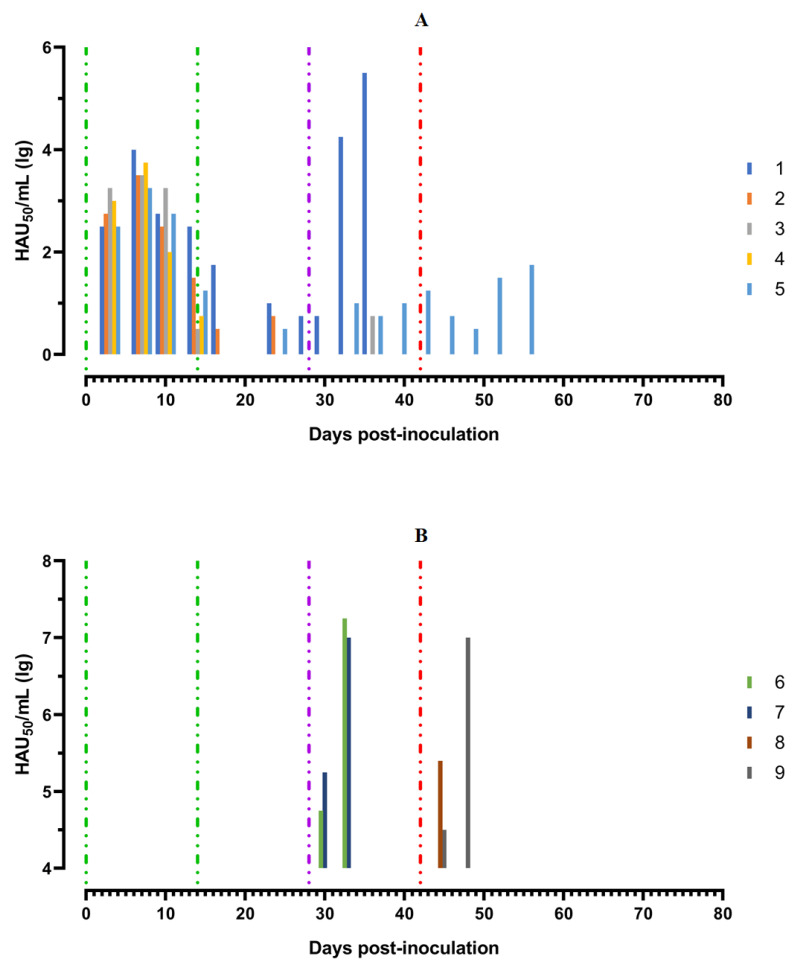
Virus titers in blood samples, obtained from pigs: (**A**) group 1 (## 1–5) inoculated with 10^6,0^ HAU_50_ ASFV–Katanga-350 and challenged at a dose of 10^3.0^ HAU_50_ ASFV–Lisbon-57 and ASFV–Stavropol 08/01; (**B**) group 2 challenged with ASFV–Lisbon-57 (## 6, 7) and group 3 challenged with ASFV–Stavropol 08/01 (## 8, 9). Each bar represents an individual animal’s values. Vertical dashed lines: green—days of inoculation of ASFV–Katanga-350, purple—challenge by ASFV–Lisbon-57, red—infected by ASFV–Stavropol 08/01 strain.

After the second inoculation, all five pigs had a normal body temperature (Figure 3A). Starting from the 22nd day post inoculation, pig #1 had an inflammation of the elbow joint of the anterior right limb, depression, decreased appetite and dyspnea (Figure 4A). The level of viremia in pigs ##1–5 was 10^2.50^–10^3.25^ HAU_50_/mL on the 5th dpi, 10^3.25^−10^4.00^ HAU_50_/mL on the 8th dpi, and 10^0.50^–10^2.50^ HAU_50_/mL on the 14th dpi. After the second inoculation, the viremia level in the period from 14 to 28 dpi was 10^0.50^–10^1.00^ HAU_50_/mL (Figure 5A).

**Figure 6:**
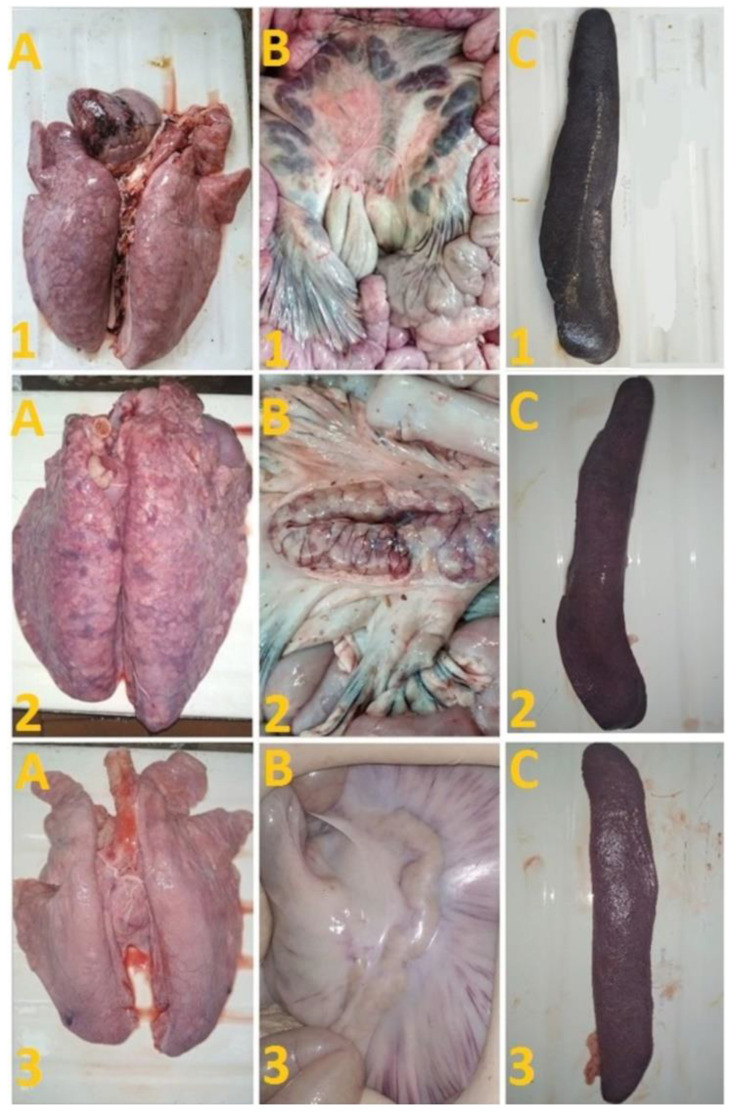
Lungs (**A**), mesenterial lymph nodes (**B**) and spleen (**C**) of pigs: # 1 with an acute course caused by inoculation with Katanga-350, followed by a challenge with Lisbon-57; pig # 2 with chronic form caused by inoculation with Katanga-350, and followed by a combined challenge with Lisbon-57 and Stavropol 08/01 strains; asymptomatic course of ASF (pig # 3) caused by inoculation with Katanga-350, and followed by a challenge with Lisbon-57 and Stavropol 08/01 strains respectively.

After challenge of pigs from groups 1 (## 1–5) and 2 (## 6, 7) by the virulent Lisbon-57 strain, the animals #1, #6 and # 7 had the following typical clinical signs of acute form of the disease: depression, feed refusal, cyanosis of the ears (Figure 4A,B). From the 3rd to 9th dpc-L they had hyperthermia (40.6–41.1) °C (Figure 3A,B). Pigs # 1 from group 1 and ## 6, 7 from group 2 had a viremia level of 10^4.25^–10^5.25^ HAU_50_/mL at 3 dpc-L (31 dpi)—and 10^5.50^–10^7.25^ HAU_50_/mL at 7 dpc-L (35 dpi) (Figure 5A,B). On the 6th–9th dpc-L, these pigs were euthanized in the preagonal state. The necropsy revealed the following pathoanatomical changes typical of ASF: serous-hemorrhagic lymphadenitis of the submandibular and inguinal lymph nodes; hemorrhagic lesions of the mediastinal, gastric, portal and mesenteric lymph nodes; hemorrhagic tonsillitis of the palatine tonsils; congestive hyperemia and pulmonary edema; hemorrhagic splenitis; and petechial hemorrhages in the kidneys and heart (Figure 6).

In the period from 1 to 14 dpc-L (29–42 dpi), pigs ## 2–4 from group 1 remained clinically healthy. In the period 13–28 dpc-L (41–56 dpi), pig # 5 from group 1 showed the following clinical signs of chronic ASF: hyperthermia (40.2–41.3) °C, depression, feed refusal followed by exhaustion, and respiratory distress (Figure 3A and Figure 4A). At 28 dpc-L (56 dpi), the animal was euthanized. On autopsy, serous hemorrhagic lymphadenitis of the submandibular, mediastinal and mesenteric lymph nodes, congestive hyperemia of the liver and kidneys, congestion and pulmonary edema, and spleen hyperplasia were recorded (data not shown). In the period 1–14 dpc-L (29–42 dpi), the viremia level in pigs ## 2–5 from group 1 did not exceed 10^0.75^–10^1.75^ HAU_50_/mL (Figure 5A).

On the 42nd dpi, pigs ## 2–4 from group 1 and ## 8, 9 from group 3 were challenged with a virulent ASF virus strain, Stavropol 08/01. As expected, non-immunized controls exhibited acute clinical signs and died or were euthanized at 5–7 dpc-St (Figure 3B and Figure 4B). The autopsy showed the following pathoanatomical changes typical of acute and super acute forms of the disease: serous-hemorrhagic exudate in the chest and abdominal cavities, hemorrhagic lymphadenitis of the submandibular, mediastinal, mesenteric, inguinal, gastric and portal lymph nodes, petechial hemorrhages in the kidneys and endocardium, and hemorrhagic splenitis.

Animals # 8 and # 9 from group 3 had a viremia level of 10^2.50^–10^3.25^ HAU_50_/mL on the 3rd dpc-St (45 dpi) in titers, and a maximum viremia level of 10^6.50^–10^7.00^ HAU_50_/mL on the 7th dpc-St (49 dpi) (Figure 5 B). From the 23rd dpc-St (65 dpi) in pig # 2, a body temperature of 40.2 °C, with its subsequent fluctuations between 39.5 and 40.8 °C, was recorded (Figure 3A). Animals # 3 and # 4 remained clinically healthy in the period 1–36 dpc-St (43–78 dpi). After infection of pigs ## 2–4 from group 1 with the Stavropol 08/01 strain, no infectious virus was detected in their blood samples (Figure 5A).

On the 78th dpi, pigs ## 2–4 were euthanized. The autopsy of animals # 3 and # 4 demonstrated no pathoanatomical changes in their internal organs (Figure 6). At the same time, the autopsy of pig # 2 showed the presence of lesions in the lungs, lobar pneumonia and edema, congestion in the liver, serous-hemorrhagic lymphadenitis of the mesenteric lymph nodes, enterocolitis, and spleen hyperplasia (Figure 6). The virus was not detected by hemadsorption test in 10% suspensions of organs (spleen, lungs, lymph nodes and tonsils) of pigs ## 3, 4, while in pigs ## 1, 2, 5–9 these organ suspensions tested positive (not shown).

### 3.4. Detection of the Viral DNA

**Figure 7:**
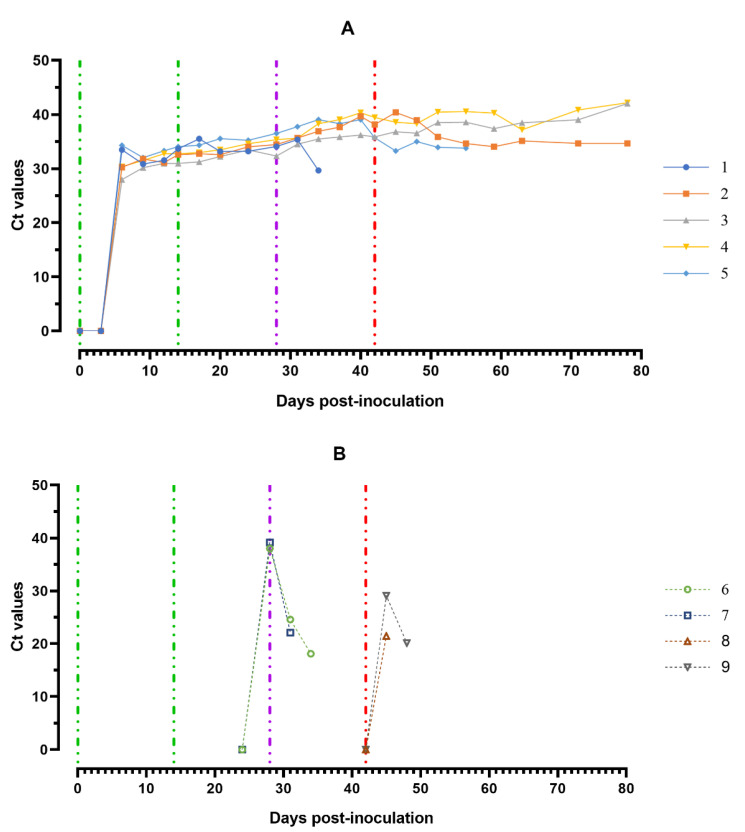
Values of ASFV-specific qPCR results of blood samples of pigs from 0 to 78 dpi: (**A**) group 1 (## 1–5) inoculated with 10^6.0^ HAU_50_ ASFV-Katanga-350 and challenged at a dose of 10^3.0^ HAU_50_ ASFV–Lisbon-57 and ASFV–Stavropol 08/01; (**B**) group 2 challenged with ASFV–Lisbon-57 (## 6, 7) and group 3 challenged with ASFV–Stavropol 08/01 (## 8, 9). Each bar represents an individual animal’s values. Vertical dashed lines: green—days of inoculation of ASFV–Katanga-350, purple—challenge by ASFV–Lisbon-57, red—infected by the ASFV–Stavropol 08/01 strain. Results are displayed as Ct.

The ASFV DNA was found in blood samples from all experimentally infected animals over the course of the trial. First individual positive qPCR results obtained from the blood samples correlated mainly to the onset of clinical signs/fever and the viable virus (Figure 7A).

The highest ASFV DNA loads in blood samples were detected at 5–8 dpi (Ct between 27.98–32.04). Throughout the experiment, clinically healthy pigs (# 3 and # 4) from group 1 showed no new peaks of Ct values, even after challenge with ASFV–Lisbon-57 and ASFV–Stavropol 08/01 strains. An increase in this parameter was detected in samples from pig # 2 and # 5 in the period 51–78 dpi (9–36 dpi-S) (Ct 34.06–35.85) and 42–56 dpi (0–14 dpi-L) (Ct 33.28–35.68) respectively. The highest values of the Ct (18.11–22.09) were reached a day before euthanasia in naïve pigs from groups 2 and 3 infected by ASFV–Lisbon-57 or ASFV–Stavropol 08/01 (Figure 7B).

**Figure 8:**
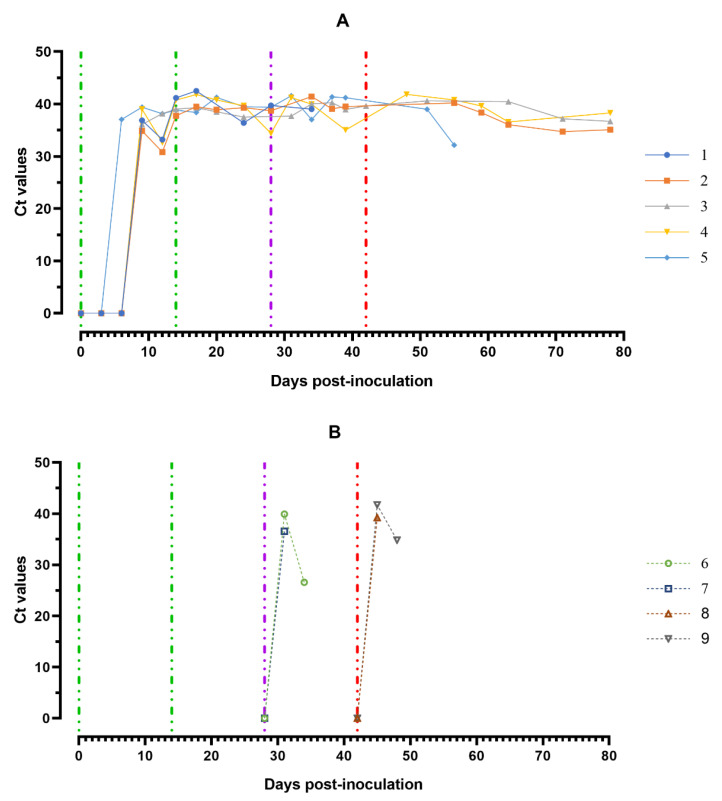
Values of ASFV-specific qPCR results of oral fluids samples of pigs from 0 to 78 dpi: (**A**) group 1 (## 1–5) inoculated with 10^6.0^ HAU_50_ ASFV–Katanga-350 and challenged at a dose of 10^3.0^ HAU_50_ ASFV–Lisbon-57 and ASFV–Stavropol 08/01; (**B**) group 2 challenged with ASFV–Lisbon-57 (## 6, 7) and group 3 challenged with ASFV–Stavropol 08/01 (## 8, 9). Each curve represents an individual animal’s values. Vertical dashed lines: green—days of inoculation of ASFV–Katanga-350, purple—challenge by ASFV–Lisbon-57, red—infected by ASFV–Stavropol 08/01 strain. Results are displayed as Ct values.

The highest ASFV DNA loads were found in oral fluids sampled from pigs from group 1 at 8–11 dpi and a day before euthanasia (#2 and # 5) (Ct 30.82–35.07), as well as from pigs from groups 2 and 3 a day before euthanasia (Ct 26.61–28.91) (Figure 8A,B).

Fluctuations of Ct values in saliva samples were most likely associated with the quality of sampling. RT PCR revealed negative results for 10% suspension samples of spleen, lungs, lymph nodes and tonsils of pigs # 3 and # 4, while the same organ suspension samples from pigs ## 1, 2, 5–9 were positive (results not shown).

### 3.5. Antibody Response in Host Animals

**Figure 9:**
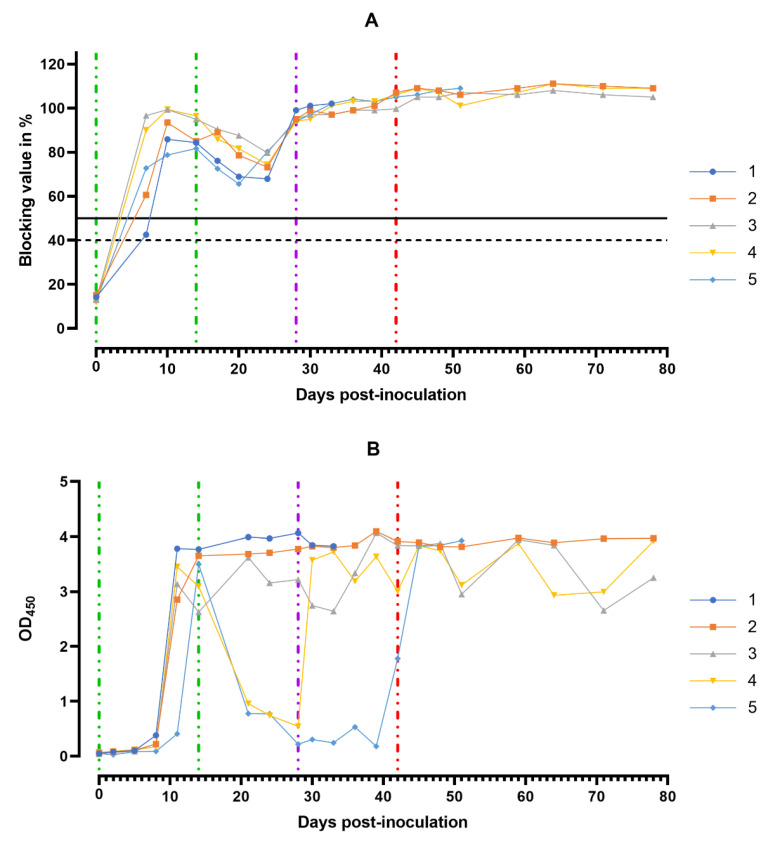
Anti-ASFV antibody response. Individual kinetics of ASFV-specific antibodies in serum samples (**A**) and in oral fluids (**B**) from pig group 1 (## 1–5) inoculated with 10^6.0^ HAU_50_ ASFV–Katanga-350 and challenged at a dose of 10^3.0^ HAU_50_ ASFV–Lisbon-57 and ASFV–Stavropol 08/01. Serum samples with a blocking percentage ≥ 50% were considered positive. Vertical dashed lines: green—days of inoculation of ASFV–Katanga-350, purple—challenge by ASFV–Lisbon-57, red—infected by the ASFV–Stavropol 08/01 strain.

ASF virus-specific antibodies in the sera samples of animals from group 1 were detectable starting from the 8th dpi, with the maximum number of antibodies detectable between 11 and 14 days (Figure 9A).

Over the next 10 days (15–24 dpi) after the second inoculation of the strain Katanga-350, a slight decrease in the levels of virus-specific antibodies was observed. By the 28th dpi, the levels of the virus-specific antibodies had reached their maximum values again, persisting up to the 78th dpi (Figure 9A). The lowest levels of the virus-specific antibodies in the sera samples were observed in pigs # 1 and # 5 in the period from 8 to 24 dpi.

In the saliva samples of pigs from group 1, high levels of the virus-specific antibodies were recorded starting from the 11th dpi until euthanasia (Figure 9B).

Naïve pigs infected with either the ASFV–Lisbon-57 strain or the ASFV–Stavropol 08/01 strain were not tested for virus-specific antibodies as they all died between days 5 and 9 post inoculation (data not shown).

## 4. Discussion

We have demonstrated in this study that experimental double inoculation of pigs with a non-virulent ASFV genotype-I, seroimmunotype-I strain, Katanga-350, can induce protective immunity in 80% of European domestic pigs against the homologous virulent European strain of ASFV, Lisbon-57. At least 50% of animals that survived were protected from subsequent challenge with a heterologous virulent European isolate of ASFV, Stavropol 08/01, belonging to genotype II and seroimmunotype VIII.

Previously, we demonstrated that CD2v/C-type lectin genotyping provides a simple approach to group ASFVs by their serotype [35]. Phylogenetic analysis based on these gene sequences revealed that the ASFV strain, Katanga-350, was grouped closely and reliably in the tree with the ASFV reference strain, Lisbon-57 (GenBank #KM609344.1), and other strains of serotype 1. Gene sequences were confirmed by serotyping of ASFV strains, Katanga-350 and Lisbon-57, by results of HAdI. Finally, the seroimmunotype identity of the Katanga-350 and Lisbon-57 ASFV strains was confirmed by immunological tests on pigs. Four (## 2–5) of five pigs inoculated with the ASFV strain, Katanga-350, were conferred with protection against challenge with the ASFV strain, Lisbon-57.

Protective immunity develops in pigs who survive viral infection with moderately virulent or attenuated variants of ASFV, with long-term resistance to homologous, but rarely to heterologous, virus challenge [43,44,45,46]. For example, pigs infected with OUR T88/3 or OUR T88/4 non-pathogenic, non-HAD virus isolate were protected against challenge with the pathogenic HAD virus (OUR T88/1), which was isolated from the same farm. Less effective protection was achieved when recovered pigs were challenged with the more distantly related Lisbon-57 isolate [47]. All isolates mentioned above belong to genotype I. However, according to the nucleotide analysis of CD2v/C-type protein genes, the OUR T88/1, OUR T88/3 and OUR T88/4 isolates belong to serotype IV, while the Lisbon-57 strain belongs to serotype I [35]. The death of pigs inoculated with OUR T88/3 or OUR T88/4 isolate after challenge with the strain, Lisbon-57, can be explained by the seroimmunological features of these strains [47]. In our work, both the attenuated strain, Katanga-350, and the virulent strain, Lisbon-57, belong to seroimmunotype I. This explains the protection of 80% of vaccinated pigs.

It was earlier reported that pigs, after sequential inoculation with homologous, attenuated and virulent strains of the ASF virus, were partially protected from subsequent challenge with heterologous isolates [47]. In our work, three group 1 pigs that survived after sequential inoculation with homologous ASFV strains, Katanga-350 and Lisbon-57, were infected with the heterologous strain, Stavropol 08/01. Two of these pigs remained healthy until the end of the observation period (78 dpi, 50 dpc-L, 36 dpc-St), while one showed clinical signs of chronic disease on the 23rd dpc-St (65 dpi, 37 dpc-L). We assume that heterotypic immunological protection will be based on the development of the mechanism of antibody-dependent cellular cytotoxicity.

Similar results were obtained in experiments on immunization of pigs with the non-virulent OUR T88/3 genotype-I isolate from Portugal and the closely related virulent OUR T88/1 genotype-I isolate. These isolates could confer protection against challenge with virulent isolates from Africa, including the genotype-I Benin 97/1 isolate and the genotype-X Uganda 1965 isolate. This immunization strategy protected most of the pigs challenged with either the Benin or Uganda isolate from both the disease and viremia. Cross-protection was correlated with the ability of different ASFV isolates to stimulate immune lymphocytes in pigs immunized by OUR T88/3 and OUR T88/1 [46].

Animals that survive consistent infection with isolates (strains) of the ASF virus of different seroimmunotypes can become sources and spreaders of mixed ASF virus populations. This mechanism seems to be one reason for the formation of heterogeneous isolates from the seroimmunotype groups X and XI [25,35].

In some studies, all animals infected with an attenuated strain developed a high antibody response after a week, which was maintained until the end of the experiment (longer than 4 months) [47,48]. We obtained similar results. The decreasing trend of the level of virus-specific antibodies in the blood sera of animals from group 1 after the second inoculation with the Katanga-350 strain in the period from 17 to 24 dpi is worthy of note. Apparently, maintaining a high level of virus-specific antibodies in blood sera until the end of the experiment suggests persistence of the virus in pigs, although the results of RT-PCR and detection of viremia in pigs # 3 and # 4 after 56 dpi showed the absence of ASFV in the blood.

Virus-specific antibodies were detected in the saliva samples of pigs from group 1 from the 11th dpi to the 78th dpi. Similar results were obtained previously with naturally attenuated and laboratory-attenuated ASFV strains [49,50]. The identified individual differences in the dynamics of the level of antibodies in saliva samples give no reason for speculation about the prognosis of the outcome after infection. The facts of long-term detection of virus-specific antibodies in saliva samples confirm the importance of ELISA for the detection of ASFV-contacted pigs among clinically and pathoanatomically healthy pigs.

In summary, we have demonstrated that, according to the sequences of the EP153R and EP402R genes and the results of the HAdI and immunological tests, this attenuated strain, Katanga-350, belongs to seroimmunotype I. Inoculation of the Katanga-350 strain resulted in transient hyperthermia. As a result, 80% of pigs were protected from the virulent strain of the ASFV seroimmunotype I, Lisbon-57. Our studies confirm that there is a partial cross-protection between ASF viruses belonging to different seroimmunotypes. This is important for the development and use of vaccines in regions where ASF is endemic. For example, in southeast Africa. The Katanga-350 strain can be considered as a basis for the development of a more advanced live recombinant vaccine against the ASF virus seroimmunotype I.

## Figures and Tables

**Table 1:** Serotyping of ASFV strains in HAdI.

Strains and Controls	Seroimmuno-Type	Serotype of Serum									Virus Control
		1	2	3	4	5	6	7	8	9	
**Lisbon-57**	**I**	−*	+**	+	+	+	+	+	+	+	+
Congo-49	II	+	−	+	+	+	+	+	+	+	+
Mozambique–78	III	+	+	−	+	+	+	+	+	+	+
France-32	IV	+	+	+	−	+	+	+	+	+	+
TSP-80	V	+	+	+	+	−	+	+	+	+	+
TS-7	VI	+	+	+	+	+	−	+	+	+	+
Uganda	VII	+	+	+	+	+	+	−	+	+	+
Rhodesia	VIII	+	+	+	+	+	+	+	−	+	+
Davis	IX	+	+	+	+	+	+	+	+	−	+
Katanga	I	−	+	+	+	+	+	+	+	+	+
Katanga-350	I	−	+	+	+	+	+	+	+	+	+
Stavropol 08/01	VIII	+	+	+	+	+	+	+	−	+	+
Serum controls	n/a −	−	−	−	−	−	−	−	−	−	−
Cell culture control	n/a −	−	−	−	−	−	−	−	−	−	−

Note: −*: absence of cells with specific hemadsorption; +**: 2–5 cells with specific hemadsorption in the field of view of the microscope. n/a—not applicable.

**Table 2:** Design of the experiment and consequences of inoculation by Katana-350 followed by challenging with Lisbon-57 and Stavropol 08/01 strains.

Group	Pig No.	Time, dpi						
		0	14	28	29–41	42	43–77	78
1	1	Katanga-350	Katanga-350	Lisbon-57	Death day 36			
	2	Katanga-350	Katanga-350	Lisbon-57		Stavropol 08/01	Chronic ASF 71–77 dpi	Euthanasia
	3	Katanga-350	Katanga-350	Lisbon-57		Stavropol 08/01		Euthanasia
	4	Katanga-350	Katanga-350	Lisbon-57		Stavropol 08/01		Euthanasia
	5	Katanga-350	Katanga-350	Lisbon-57		Chronic form ASF day 41–56	Euthanasia day 56	
2	6			Lisbon-57	Death day 34			
	7			Lisbon-57	Death day 37			
3	8					Stavropol 08/01	Death day 50	
	9					Stavropol 08/01	Death day 50	

## Data Availability

The data presented in this study are available on request from the corresponding author.
